# Impact of COVID-19 on pneumonia-focused antibiotic use at an academic medical center

**DOI:** 10.1017/ice.2020.362

**Published:** 2020-07-23

**Authors:** Matthew J. Nestler, Emily Godbout, Kimberly Lee, Jihye Kim, Andrew J. Noda, Perry Taylor, Rachel Pryor, J. Daniel Markley, Michelle Doll, Gonzalo Bearman, Michael P. Stevens

**Affiliations:** 1 Virginia Commonwealth University School of Medicine, Richmond, Virginia; 2 Healthcare Infection Prevention Program. Virginia Commonwealth University Health System, Richmond, Virginia; 3 Virginia Commonwealth University Health System, Richmond, Virginia; 4 Hunter Holmes McGuire VA Medical Center, Richmond, Virginia

*To the Editor—*Optimizing antimicrobial use and stewardship during the global spread of severe acute respiratory coronavirus virus 2 (SARS-CoV-2) is an important goal for health systems. A review published in May 2020 found that only 8% of patients with coronavirus disease 2019 (COVID-19) had a bacterial or fungal coinfection, while 72% of patients received antimicrobial therapy.^1^ Many patients requiring hospitalization for COVID-19 present with symptoms mimicking community-acquired bacterial pneumonia prompting empiric antibiotic use.^2^ High antibiotic use might also stem from provider experience with hospitalized influenza patients of which 11%–35% may have a bacterial superinfection.^2,3^ Antibiotic usage trends are starting to be published; a study by Velasco Arnaiz et al^4^ showed increased pediatric inpatient azithromycin and ceftriaxone use in March and April of 2020 compared to the same months in 2019.

We examined inpatient pneumonia-focused antibiotic use trends at Virginia Commonwealth University (VCU) Health System, an 865-bed urban academic medical center. We hypothesized that antibiotic days of therapy per 1,000 patient days (DOT per 1,000 PD) for key antimicrobials targeting pneumonia would be affected for April and May of 2020 when compared to the average DOT per 1,000 PD over the preceding year due to the impact of COVID-19 on our health system.

## Methods

The antibiotics ceftriaxone, azithromycin, levofloxacin, doxycycline, cefepime, piperacillin-tazobactam, meropenem, and vancomycin were chosen due to their common use for either community-acquired pneumonia (CAP) or hospital-acquired/ventilator-associated pneumonia (HAP/VAP) coverage. Antibiotic DOT per 1,000 PDs were examined for 3 units: a medical intensive care unit (MICU), a coronary intensive care unit (CICU), and a progressive medicine unit. The percentages of COVID-19–positive patient days were calculated for each unit by month. For each unit, the normality of the April 2019–March 2020 monthly data were confirmed using a histogram and kurtosis or skewness scores. Seasonality was also checked via graph and determined to not be a substantial influence on the data. A 2-sample *t* test assuming equal variances was performed with the first group being the April 2019–March 2020 monthly data and the second being April or May 2020. Thus, we tested the null hypothesis that antibiotic use in April or May 2020 was the same as the mean use from April 2019 to March 2020. The 2-tailed *P* values are reported in Table 1 and P ≤ .05 was considered significant. The analyses were conducted using Excel version 2002 software (Microsoft, Redmond, WA).

**Table 1.** Antibiotic Use for April and May 2020 Versus April 2019–March 2020

Unit	April COVID-19 PD, No. (%)	May COVID-19 PD, No. (%)	Antibiotic			April 2020 vs Mean		May 2020 vs Mean
				April 2019– March 2020, Mean DOT /1,000 PD	April 2020, DOT /1,000 PD	*P* Value	May 2020, DOT /1,000 PD	*P* Value
MICU	156 (28)	212 (30)	Cefepime	134	117	.61	184	.16
			Pip-Tazo	341	385	.42	324	.75
			Meropenem	72	78	.81	56	.49
			Vancomycin	281	262	.55	271	.76
			Ceftriaxone	55	193	.00	81	.10
			Azithromycin	50	109	.03	49	.95
			Levofloxacin	56	24	.07	3	.01
			Doxycycline	15	12	.81	0	.23
CICU	6 (3)	14 (5)	Cefepime	53	72	.56	46	.84
			Pip-Tazo	210	216	.89	268	.25
			Meropenem	25	38	.42	46	.20
			Vancomycin	167	168	.95	168	.95
			Ceftriaxone	31	131	.00	36	.79
			Azithromycin	14	17	.80	31	.26
			Levofloxacin	9	14	.57	0	.31
			Doxycycline	18	21	.86	0	.19
PM	280 (64)	304 (69)	Cefepime	49	32	.35	35	.45
			Pip-Tazo	132	95	.09	94	.08
			Meropenem	16	10	.44	26	.30
			Vancomycin	97	72	.39	76	.47
			Ceftriaxone	59	138	.00	87	.19
			Azithromycin	27	103	.00	60	.07
			Levofloxacin	26	10	.08	5	.03
			Doxycycline	10	0	.38	0	.38

Note. MICU, medical intensive care unit; CICU, cardiac intensive care unit; PM, progressive medicine unit; Pip-Tazo, piperacillin-tazobactam; PD, patient days; DOT, days of therapy.

## Results

We detected a significant increase in April 2020 ceftriaxone use in the MICU (*P* < .001), the CICU (*P ≤* .001), and the progressive medicine unit (*P* = .0024) as well as April 2020 azithromycin use in the MICU (*P* = .031) and PM (*P* < .001). There was a significant decrease for May 2020 levofloxacin use in the MICU (*P* = .0066) and the progressive medicine unit (*P* = .029) (Table 1).

## Discussion

All 3 units demonstrated a significant increase in ceftriaxone use in April 2020. The MICU and the progressive medicine unit also demonstrated increased azithromycin use in April 2020. Notably, azithromycin use did not significantly increase in the CICU (perhaps related to a greater concern for risk for cardiac toxicity from this drug). Ceftriaxone and azithromycin are commonly used for community-acquired pneumonia, and we suspect that their use increased to empirically cover bacterial superinfection in patients who were suspected of having COVID-19. Interestingly, the April and May use patterns appeared to be independent of unit COVID-19 patient days (Table 1). Our hospital began testing all patients for SARS-CoV-2 on admission to the hospital on April 27, which may explain the reversion to baseline usage from April to May, especially in the CICU, where the total percentage of COVID-19–positive patients remained low. Our MICU is a closed unit with a limited number of attending providers, and patients with COVID-19 in the progressive medicine unit were mostly cared for by our hospital medicine group. Possibly, these 2 respective groups developed experience with managing these patients over the course of April and this impacted the reversion in antibiotic use trends. More research is needed to more fully understand these use trends.

There was no significant increase in the use of antipseudomonal β-lactams (ie, cefepime, piperacillin-tazobactam, and meropenem) or vancomycin across the units studied. This finding suggests that clinicians were empirically using CAP-focused antibiotics in April 2020 (with the exception of the CICU with azithromycin) as opposed to empirically giving HAP- or VAP-focused antibiotics. Our hospital has a longstanding and aggressive antimicrobial stewardship program that has published CAP and HAP/VAP guidelines. We suspect that these guidelines helped limit the use of HAP/VAP-focused antibiotic coverage in April; HAP is defined in our guidelines as occurring ≥48 hours after admission with pneumonia not present at the time of admission. Additionally, meropenem is restricted at VCU Health. The decrease in levofloxacin use in the MICU and PM units during May 2020 is not well understood and warrants further study.

This analysis has several limitations. Because it was conducted at a single medical center, our results may not be generalizable. Additionally, our vancomycin use data include both IV and oral formulations, although we think the impact of this factor on our data is very low because IV administration is predominant at our hospital.

The COVID-19 pandemic has dramatically impacted health systems, and concern that antibiotic use may drive antibiotic resistance is widespread. Our results indicate an initial uptick in CAP-focused empiric antibiotic use with a subsequent reversion to baseline use. Notably, we did not see a significant increase in the use of antipseudomonal β-lactam antibiotics or vancomycin. The roles of active antimicrobial stewardship, local treatment protocols, and universal COVID-19 testing on antibiotic use all warrant further study.
